# Molecular Dynamics Simulations of Essential Oil Ingredients Associated with Hyperbranched Polymer Drug Carriers

**DOI:** 10.3390/polym14091762

**Published:** 2022-04-26

**Authors:** Vasilios Raptis, Kostas Karatasos

**Affiliations:** 1 Chemical Engineering Department, Aristotle University of Thessaloniki, 54124 Thessaloniki, Greece; 2 Theoretical and Physical Chemistry Institute, National Hellenic Research Foundation, 11635 Athens, Greece

**Keywords:** hyperbranched polymers, molecular dynamics, drug carrier, essential oil

## Abstract

Our work concerns the study of four candidate drug compounds of the terpenoid family, found as essential oil ingredients in species of the Greek endemic flora, namely carvacrol, p-cymene, γ-terpinene, and thymol, via the simulation method of molecular dynamics. Aquatic solutions of each compound, as well as a solution of all four together in realistic (experimental) proportions, are simulated at atmospheric pressure and 37 °C using an OPLS force field combined with TIP3P water. As verified, all four compounds exhibit a strong tendency to phase-separate, thereby calling for the use of carrier molecules as aids for the drug to circulate in the blood and enter the cells. Systems of two such carrier molecules, the hyperbranched poly(ethylene imine) (HBPEI) polyelectrolyte and hyperbranched polyglycerol (HPG), are examined in mixtures with carvacrol, the most abundant among the four compounds, at a range of concentrations, as well as with all four compounds present in natural proportions. Although a tendency of the terpenoids to cluster separately persists at high concentrations, promising association effects are observed for all drug–polymer ratios. HBPEI systems tend to form diffuse structures comprising small mixed clusters as well as freely floating polymer and essential oil molecules, a finding attributed to the polymer–polymer electrostatic repulsions, which here are only partially screened by the counterions. On the other hand, the electrically neutral HPG molecules cluster together with essential oil species to form a single nanodroplet. Currently, terpenoid–polymer clusters near lipid bilayer membranes are being studied to determine the propensity of the formed complexes to enter cell membranes.

## 1. Introduction

Candidate drug compounds must fulfill certain criteria prior to administering them for trial or actual medical purposes [1]. Usually, such a compound will be a non-toxic organic molecule expected to alter the function of particular proteins involved in a disease, thanks to the molecule’s small size and suitable shape, thereby bringing about a therapeutic effect. Ideally, the molecule would be incapable of activating or inhibiting proteins other than the target, otherwise undesirable side effects may arise. It would also need to be stable enough to be taken up by the human body without decomposing and to reach its biological target. Its properties should allow it to be assimilated at a fast rate, be transferred by the blood circulatory system, and enter the cells wherein the therapeutic action will take place.

Rational drug discovery and design processes consist of several steps, including the screening of very large libraries of compounds to find ones that could bind to a target protein, which are then subjected to a set of restrictions (low molecular weight, limited number of hydrogen bond donors and acceptors, an upper bound of lipophilicity expressed as the octanol–water partition coefficient, and so on) [2]. On top of the efforts to explore the vast chemical field of organic compounds, a number of experimental steps are taken to ensure safety, efficacy, and minimal size effects prior to approving a new drug, resulting in a lengthy process (up to several years [3]) that requires huge investments (of the order of billions of euros) [4].

To avoid such a costly and time-consuming process, alternative routes have been adopted by the drug industry. One of them relies on so-called drug repurposing, i.e., limiting the search for new drugs to the chemical subspace of compounds developed and used in the past for other illnesses, which are known to have the useful properties required of a drug [5]. Such is the case, for instance, of the infamous thalidomide, which turned out to be highly effective in the treatment of several diseases, such as leprosy and plasma cell myeloma, as long as it is restricted to patients who do not try to conceive a child in order to avoid the risk of teratogenic birth defects [6].

Another interesting approach involves the use of natural ingredients that can be found in plants, whether extracted or synthesized in the laboratory [7,8]. A variety of plants have been used since antiquity to contribute to the treatment and cure of various illnesses, as well as to overall well-being. This is not unsound scientifically, as the human body possesses protein receptors such as opioids and cannabinoids that can be activated by a range of natural compounds [9,10]. Natural products directly extracted from plants are complex mixtures of a large array of similar species, such as terpenoids, which can have synergistic health effects [11]. Such products vary from region to region, depending on the diversity of the endemic flora. The development of novel drugs derived from native plants with advantageous health effects can be of particular interest to the local economy and overall community welfare, while enhancing the options for new successful medicines worldwide.

Natural compounds in isolation from their original source may lack some of their ability to conform to the criteria that define a suitable drug, especially with regards to administration and uptake by the body. For instance, oral administration in the form of aquatic solutions may be complicated due to the compounds’ tendency to phase-separate—this is also the case for novel drugs in general. So-called drug carriers that consist of innocuous, biocompatible species can improve the pharmacokinetic properties of a given compound by enhancing its circulation through the body, while at the same time allowing fast release at the drug’s target. Among other options (liposomes, amphiphilic micelles, nanoparticles, and so on), dendrimers and hyperbranched polymers can be attractive as drug carriers [12,13].

A hyperbranched species with a certain amphiphilic character could be capable of associating with small lipophilic organic species and forming water-soluble nanodroplets that can dissolve in the mostly aquatic environment of the blood. When the nanodroplets reach the cell membrane they will dissolve in it, thanks to the drug’s lipophilicity, as well as the polymer’s lipophilic groups, thereby ensuring fast and efficient release [14]. The ability of a hyperbranched polymer to act as a drug carrier depends on a number of factors, such as its molecular weight, the presence of hydrophilic and hydrophobic groups, the strength of binding with the drug compound, and the nature of the interactions with the cell membrane. Understanding the particular interactions and microscopic mechanisms underlying pharmacokinetics may be crucial in the selection of novel drugs and their carriers. The use of molecular modeling methods can be an indispensable complement to experimental techniques when it comes to the study of drug transport and release at the molecular level, provided reliable models (force fields) are available.

Molecular modeling is an established step in drug design in the form of molecular mechanics calculations that predict whether a compound can bind successfully to the target receptor [15]. However, modeling and theoretical calculations need not be restricted in this area. Molecular simulation techniques can reliably predict many properties of candidate drug compounds, whether in isolation or in solution, alone or in the presence of other species such as potential drug carriers. The time dependence of clustering and association is an important parameter governing drug solubilization and its enhancement with the aid of drug carriers; molecular dynamics simulations can be particularly useful in this respect.

In this work, we used molecular dynamics simulations to look at the properties of four compounds of the terpenoid family that are commonly encountered in species of the Greek endemic flora [16], namely carvacrol, p-cymene, γ-terpinene, and thymol. These are small lipophilic molecules that are usually mixed with many other small organic molecules that form the broader family of essential oils. These compounds exhibit wide biological activity, including antimicrobial [17,18,19,20,21], antifungal [22,23,24], antioxidant [24], antiviral [25,26], and anticancer [27] properties. They are also considered appropriate additives to improve the quality and enhance the safety of food products [16,17,18,19,20]. Our aim was to study their interactions with two potential drug carrier compounds, namely hyperbranched polyethylene imine (HBPEI), a polyelectrolyte, and hyperbranched polyglycerol (HPG), an electrically neutral amphiphilic polymer, to determine whether they form stable clusters.

The two polymers under study fall in the class of hyperbranched polymers that are attractive drug carriers due to their biocompatibility, rich architecture, and topology, combined with their ease of synthesis as compared to their competitor dendrimers [28,29]. Zhou et al. [30] in 2010 summarized numerous applications of HBPEI and HPG in drug, multidrug, and gene delivery scenarios. The physicochemical conditions (temperature, concentration, acidity, pH) are important in drug delivery applications, as these vary within a narrow range in the human body. HBPEI has been shown to exhibit superior encapsulating properties over its linear counterpart, especially at low polymer concentrations and weakly acidic to marginally alkaline pH values [31]. Sideratou et al. [32] successfully developed a HBPEI-based temperature-triggered anticancer drug delivery agent. Gene delivery and combined drug–gene delivery are areas where polyethyleneimine of various architectures, including HBPEI, can play a promising role as a vector, as discussed by Zakeri et al. [33]. In the broader area of therapeutic agent delivery, HPG- and HBPEI-containing copolymers have been studied with respect to protein delivery or synthetic vaccine manufacturing [34,35,36].

Among the four essential oil ingredients under study, carvacrol is the most abundant, with the other three being found at significantly lower concentrations. Thus, most of our polymer–compound simulations in the present work related to systems of carvacrol over a range of concentrations. However, we have also looked at systems containing all four compounds in realistic proportions to evaluate the effect of their coexistence and possible synergy. Our calculations yielded promising results for both hyperbranched species regarding their ability to form stable clusters with the essential oil ingredients. To fully determine the two polymers’ ability to act as drug carriers, we are currently looking at polymer–compound clusters near a lipid bilayer modeling the cell membrane to determine whether the drug compound will be successfully transported and released into the membrane. These last simulations will be discussed in a forthcoming article.

The next section of the present work is dedicated to a description of the compound and compound–polymer aquatic mixtures that were simulated and the simulation details. The properties of interest are also listed and the methods to predict them by analyzing the simulation trajectories are presented. The results are reported in Section 3 and thoroughly discussed in Section 4. Our conclusions are summarized and future plans are mentioned in the last section.

## 2. Materials and Methods

### 2.1. General Considerations

**Figure 1:**
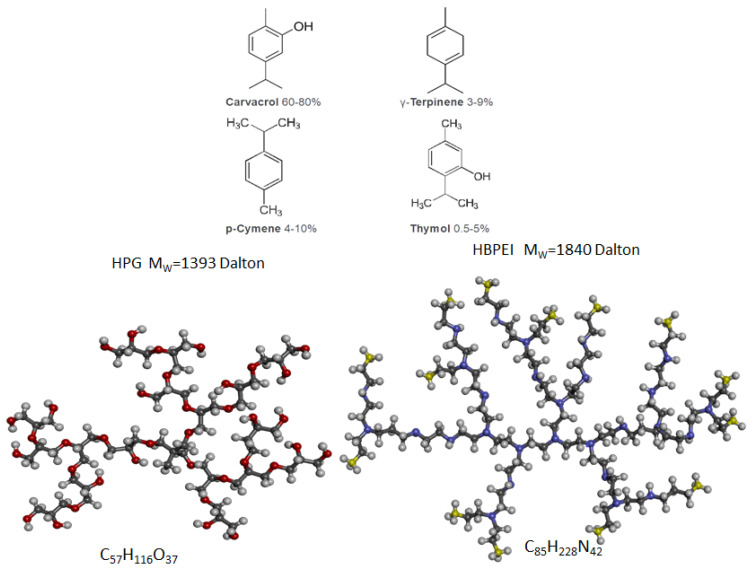
Illustration of the four essential oil ingredients under study (**top**) and candidate drug–carrier hyperbranched polymers (**bottom**). The hyperbranched polymers are shown as ball–stick representations, with hydrogen atoms shown in white, oxygen atoms in red, carbon atoms in grey, non-protonated nitrogen atoms in purple, and protonated nitrogen atoms in yellow.

In this work, we looked at aqueous solutions of the four low-molecular-weight compounds mentioned in the Introduction, heretofore collectively referred to as ‘(drug) compounds’, ‘terpenoids’, or ‘(essential oil) ingredients’, namely carvacrol, p-cymene, γ-terpinene, and thymol, whether alone or in the presence of hyperbranched polymer species that can act as association agents. These four compounds, depicted in Figure 1, are very similar in their size and structure, as they mainly consist of a six-carbon ring and an isopropyl group attached to the ring. Among them, carvacrol and thymol possess one hydroxyl group while the other two do not. Thus, we have two out of four compounds capable of hydrogen bonding, allowing them to study the systems of interest in a comparative manner and highlight the role of hydrogen bonding as compared to mere hydrophobic interactions.

As mentioned in the Introduction, carvacrol is the most prevalent compound in naturally occurring essential oil mixtures, while the other three are found in concentrations that are lower by at least an order of magnitude. For this reason, the majority of our simulations relate to aquatic solutions of carvacrol; a few simulations of aquatic solutions of the other three compounds are also reported (without association agents), as well as mixtures of all four compounds in realistic proportions (with or without association agents). The structure and composition of our compounds suggests that they should be hydrophobic, which was also confirmed by our simulations. Thus, we were interested in determining water-soluble agents that could interact with the terpenoids and solubilize them. In our simulations, we looked at the aforementioned polymer species that can form clusters with compound molecules, namely hyperbranched poly(ethylene imine) and hyperbranched polyglycerol. In the following sections, we report simulations and the results thereof of aquatic solutions of the drug compounds under study (mainly carvacrol), whether alone or in the presence of HBPEI or HPG.

### 2.2. Simulation Details

The OPLS (optimized potentials for liquid simulations) atomistic force field [37,38,39,40,41] was chosen to model compounds and polymers for all simulations reported in this work. TIP3P water [42] was the selected solvent model. The systems under study included water solutions of a single compound, a solution of all four compounds in realistic proportions, carvacrol solutions in the presence of HBPEI at various polymer–carvacrol proportions, carvacrol solutions in the presence of HPG at a range of polymer–carvacrol ratios, and two solutions of all four compounds in realistic proportions in the presence of either HBPEI or HPG. Two different system sizes were looked at in the case of HBPEI + carvacrol and HPG + carvacrol systems, namely systems with 8 or 27 polymer molecules in the simulation cell, in order to take size effects into account with regards to the clustering processes. The larger size was also adopted for compound mixtures of realistic compositions combined with polymeric species. In all systems used, the HBPEI molecules were partially ionized to mimic neutral pH conditions [32]. In these systems, an appropriate amount of counterions was also included in order to maintain the overall electrical neutrality. Details of the simulated systems (number of molecules per compound, number of atoms per molecule, duration of simulations) are summarized in Table 1 and Table 2.

Systems of compound solutions (without polymer) were built by placing replicas of carvacrol or other terpenoid molecules at random positions in a cubic simulation cell (taking care to avoid too close distances) and filling the remaining space with TIP3P water molecules. Potential energy was minimized by successively invoking the steepest descent and conjugate gradient algorithms, then the system was subjected to short isothermal–isochoric (NVT) and isothermal–isobaric (NPT) MD simulations (100 ps with a 1 fs time step) at ambient pressure and at a temperature of 310 K. These steps sufficed to eliminate any orientation correlation of the compound molecules. The output systems were then subjected to MD simulations up to 21 ns, at the isothermal–isobaric ensemble at atmospheric pressure and a temperature of 310 K, the human body temperature. It was verified that the first nanosecond was sufficient for full equilibration in terms of the system’s potential energy, including its components, simulation cell dimensions, and position and orientational decorrelations of all compound molecules. The systems containing all four compounds were constructed and simulated in the same manner.

Systems of polymer–compound solutions were generated via a more complicated procedure. First, a smaller cubic cell was constructed, with a single polymer molecule in the middle, replicas of compound molecules surrounding it such that they did not overlap with the polymer and with each other, and TIP3P water filling the remaining space. The system was equilibrated in the same way as described above for the aquatic drug compound solutions (energy minimizations followed by short NVT and NPT MD runs at 310 K). The output structure was replicated along the x, y, and z directions two or three times, resulting in 8 or 27 polymer chains and the corresponding numbers of compound and water molecules, respectively. Care was taken to rebuild the molecules crossing the faces of the small cell and properly applying cubic periodic boundary conditions at the faces of the large cell. The large simulation cell was once again subjected to the same steps of energy minimization and short NVT and NPT MD runs. The output cell was subjected to 5 ns long NPT MD simulations at ambient pressure and temperatures of 400 K, 500 K, and 600 K in order to decorrelate the systems, especially with respect to the polymer orientation and overall configuration. It was verified that the terpenoid molecules in the output structure were practically homogeneously dispersed in the system.

Simulations of the systems listed in Table 1 lasted up to 21 ns (the first ns was intended for equilibration). Systems from Table 2 (at the higher drug–polymer proportions) by removing polymer molecules were also simulated.

In Table 2, “HBPEI” denotes the presence of hyper-branched polyethyleneimide and “HPG” the presence of hyper-branched polyglycerol molecules. Numbers in parentheses in the first column indicate the polymer–carvacrol molecular proportions. Each system contains either 8 (when not mentioned) or 27 (explicitly mentioned) polymer hyperbranched chains. Simulations of the systems listed herein lasted 100 ns, except the HBPEI 27-polymer chain systems for which MD runs lasted 200 ns to ensure a lack of complete phase separation.

The resulting structure was then subjected to long NPT MD runs at ambient pressure and at a temperature of 310 K. It was confirmed that the first few nanoseconds sufficed to attain equilibration in terms of cell dimensions and potential energy.

Simulations of compounds without any polymer agent were relatively short (1 ns for equilibration purposes followed by an additional 10 or 20 ns run to carry out statistics) because they sufficed to capture the characteristic timescales in these systems. On the other hand, solutions with HBPEI or HPG required significantly longer runs, i.e., 100 ns, to ensure that polymer relaxation timescales, and more importantly clustering timescales were adequately sampled in our calculations. In the case of HBPEI, including 27-polymer molecule systems, the simulation was prolonged by an additional 100 ns (200 ns in total) to ensure that the cluster size distribution would not change, for reasons discussed in detail in the next sections.

Finally, systems of drug compound aquatic solutions have also been looked at by taking polymer–drug systems at higher drug–polymer proportions and removing the polymer molecules. All other drug and polymer–drug systems were generated ‘from scratch’ and equilibrated and simulated with the aid of the GROMACS Molecular Dynamics package and its companion tools [43,44]. Simulation results were analyzed using GROMACS companion tools and other codes mentioned explicitly in the next sections.

### 2.3. Methods of Analysis

The emphasis in this work is placed on properties that quantify various aspects of the clustering, phase separation, and association behavior of the species involved in the simulations and point to explanations of such behavior. Thus, the number and size of molecular clusters of dissolved compounds (mostly carvacrol) with time were determined via the DBSCAN (density-based spatial clustering of applications with noise) algorithm [45,46] implemented in an in-house code. These results were compared to the average number of molecules of a given species β around another molecule of species α, determined as the integral of the corresponding pair distribution function g_αβ_(r) of α, β molecular centers of mass [47] up to a cutoff distance. In our calculations, we looked at the first coordination shell defined by the distance of the minimum between the first and second peaks of the pair distribution function.

While radial distribution functions provide reliable information about the average microstructure, it is also interesting to look at spatiotemporal correlations that capture the underlying dynamics and offer insights about the microscopic mechanisms that give rise to the cluster formation, as well as the dynamic equilibrium between clusters and their environment. Self, G_s_(r,t), and distinct, G_d_(r;t), van Hove correlation functions were used for the spatiotemporal characterization of the individual and collective transport properties of the essential oil ingredients. By analogy with radial distribution functions, the products 4πr^2^G_s_(r;t) were computed to quantify the probability of particles to be displaced by r regardless of direction, within time scale t. In the same vein, mass transport time scales (also serving as a measure of equilibration) were looked at via the mean square displacement (MSD), ${\left(r\left(t+\Delta t\right)-r\left(t\right)\right)}^{2}$; the resultant self-diffusivities, D, were calculated at the hydrodynamic limit with the aid of the well-known Einstein relation [48,49]:

MSD=6Dt

Another important property, which was closely related to clustering and association processes, is the formation of hydrogen bonds (when applicable) among the various species comprising our systems. Our focus is on the polymer–polymer, compound–compound, and polymer–compound pairs in the compound–polymer systems. The numbers of such hydrogen bonds with time were recorded and their time autocorrelation function was integrated to reveal characteristic times scales regarding the tendency of a given molecule to stay in the vicinity of another molecule.

Finally, the polymer–compound binding energy was calculated with the aid of the g_mmpbsa package [50,51] in order to quantify the propensity of polymer–compound clusters to form spontaneously and to examine their thermodynamic stability. In the case of mixtures containing all four compounds of interest, these were taken as a single species, i.e., interactions were computed between the polymer and any compound, regardless of the compound type.

## 3. Results

**Figure 2:**
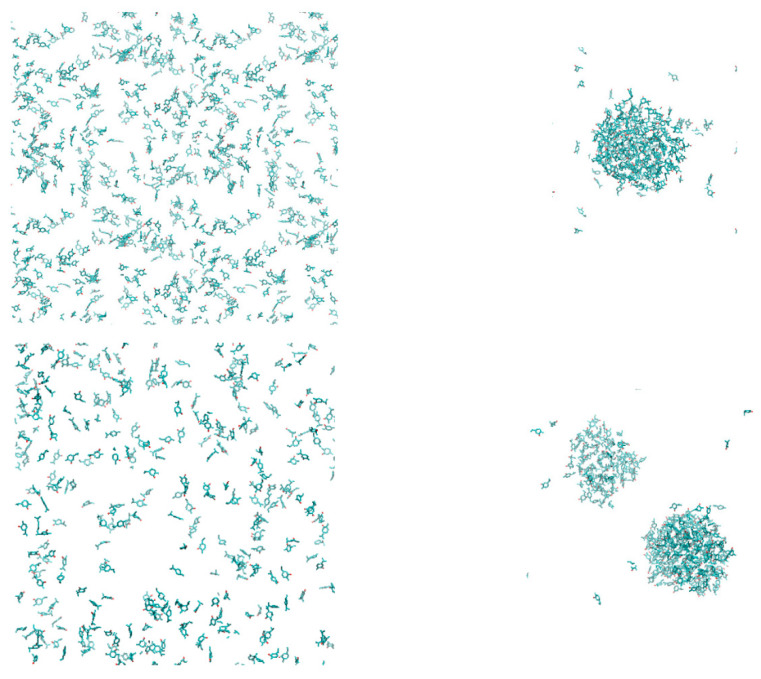
Aquatic solution of carvacrol 0.65 mol/L in its initial state (left, top) and after 6.5 ns (right, top); similar results for the terpenoid mixture in realistic proportions (0.236 mol/L carvacrol, 0.023 mol/L p-cymene, 0.019 mol/L γ-terpinene, 0.009 mol/l thymol) after 15 ns (bottom figures). Water molecules are omitted for clarity.

As mentioned in previous sections, terpenoid solutions ended up in phase separation during the first few nanoseconds of simulation. Typical examples are shown in Figure 2, showing clusters formed in aquatic solutions of carvacrol and terpenoid mixtures after 6 ns and 15 ns of simulation, respectively. Similar behavior ending in complete phase separation was observed in all other simulated terpenoid solutions. This finding was expected and confirmed the reliability of the force field. It is briefly noted that indications of equilibration (energy components, cell dimensions, molecular reorientation time scales, diffusion of essential oil ingredients) were satisfied within sub-nanosecond to nanosecond time scales, confirming that the observed phase separation represents a stable state. In the remainder of this article, only the polymer–essential oil systems will be discussed.

**Figure 3:**
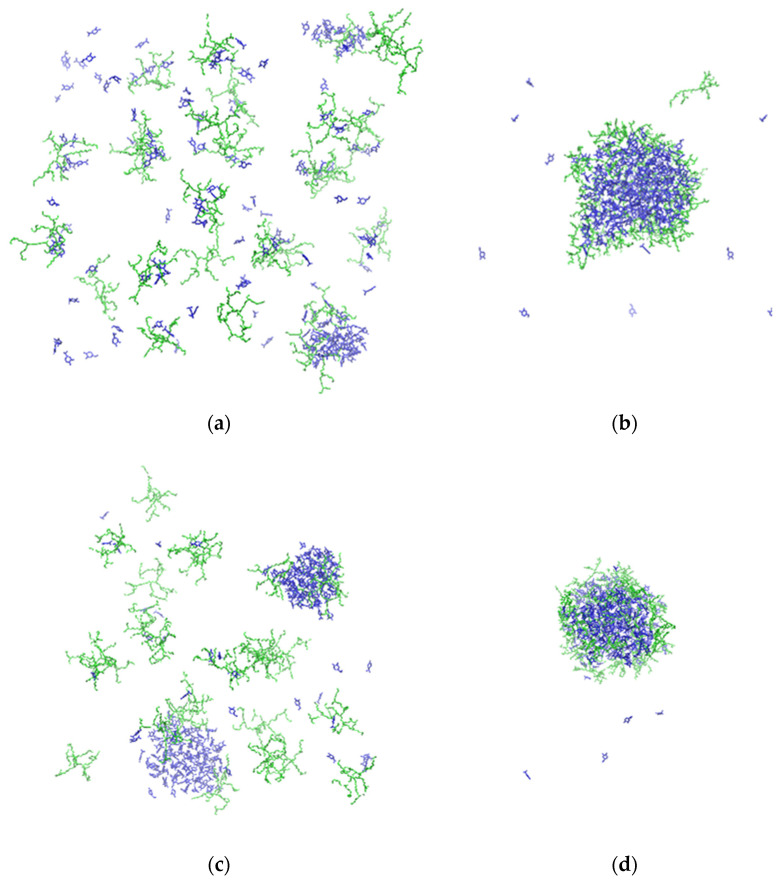
Final configurations of 27-polymer chain systems with carvacrol or four-ingredient essential oil mixtures. The numbers in parentheses denote the relative molar proportions: (**a**) HBPEI–carvacrol (1:7); (**b**) HPG–carvacrol (1:10); (**c**) HBPEI–carvacrol–p-cymene–γ-terpinene–thymol (27:189:18:15:7); (**d**) HPG–carvacrol–p-cymene–γ-terpinene–thymol (27:270:26:22:10); green: polymer; blue: essential oil molecules. Water molecules are omitted for clarity.

Visual inspection of the MD trajectories provides a clear illustration of the differences between the clustering process observed in the HPG and in the HBPEI systems. For instance, the final configurations of the 27-polymer chain systems of HPG and HBPEI (with carvacrol and with essential oil mixtures) shown in Figure 3 indicate a stark difference in the type and extent of clustering that takes place in the two kinds of systems. In HPG-containing mixtures, the essential oil molecules tend to phase-separate and form nanodroplets as they would in the absence of a polymer. However, the polymer molecules are attached on the surface of the droplet, in a manner akin to micelles. This is consistent with the hydrophobic nature of the essential oil ingredients and the amphiphilic nature of the polymer molecules.

In contrast, equilibrated configurations of HBPEI systems contain freely floating polymer and essential oil ingredient molecules coexisting with small- or medium-sized ingredient molecule clusters, as well as polymer chains either existing freely or associated with a small number of essential oil molecules in dynamic equilibrium with each other. The phase separation of essential oils is more evident in the mixture. Due to the differences observed between the final HPG and HBPEI configurations, the simulations of large (27 polymer molecules) HBPEI systems were prolonged for another 100 ns to ensure that their configurations at the end of the first 100 ns were indeed representative of a steady state rather than a transient regime. No substantial change was observed in the systems after prolonging the simulations, based on both visual inspection and the quantitative measures discussed in the next paragraphs. We attempt to explain the difference between HBPEI and HPG that these results indicate in the next section.

Post-processing of the trajectories yields additional valuable information that can be expressed in a quantitative manner. It should be noted that the results presented in this section relate to the equilibrated parts of molecular dynamics trajectories, where the properties of interest fluctuate around a constant average unless otherwise noted. The timescales at which equilibration is attained vary with the properties under discussion, and will be usually shorter for thermodynamic properties or short-range dynamics and longer for transport and clustering processes.

**Figure 4:**
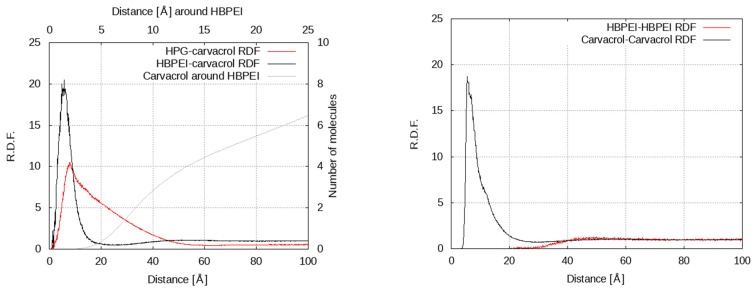
On the **left**, polymer–carvacrol radial distribution functions for 27-polymer chain systems of HBPEI–carvacrol (red) and HPG–carvacrol (black) mixtures; the number of carvacrol molecules around a HBPEI molecule (grey) is also shown for distances up to 25 Å, using different axes (upper x-axis and right y-axis). On the **right**, polymer–polymer and carvacrol–carvacrol radial distribution functions for 27-polymer chain systems of HBPEI–carvacrol.

The polymer–carvacrol center-of-mass radial distribution functions (rdf) shown in Figure 4 were calculated with the aid of a technique proposed by Theodorou and Suter [52] to extend the calculation beyond half the simulation cell length. The rdf curves were calculated over the last 50 ns of each trajectory and provide measures of the length scales that characterize the equilibrium configurations of the systems. In particular, the HPG–carvacrol radial distribution function (Figure 4 left) exhibits a peak at about 8 Å, which is consistent with the expected polymer–carvacrol center-of-mass distance based on the molecules’ dimensions when in close contact. The peak corresponds to the polymer–carvacrol pairs at the surface of the formed nanodroplet, whereas an almost linear tail at longer distances represents the carvacrol molecules deeper in the cluster at corresponding distances from its surface.

The contributions to the rdf. at distances between x and x + Δx from a polymer situated on the surface of the cluster come from the section of the cluster with the shell defined by the corresponding spheres of radii x and x + Δx. Assuming a simple spherical model of radius R for the cluster (which is clearly a crude approximation) and letting Δx → 0, this zone is a spherical cap and the polymer–drug rdf. at x, g(x), should be proportional to the cap’s surface. It is easy to verify that this surface equals:

$$A=2{\pi x}^{2}\left(1–\frac{x}{2R}\right)$$

which is maximized at x = 4R/3 before falling to zero at x = 2R. This prediction does not agree with peak characterizing the HPG–carvacrol rdf, since the maximum is located at clearly shorter a distance, given that the droplet’s radius is on average about 3 nm. Although this discrepancy can certainly be attributed in part to the cluster’s deviation from a spherical shape, it may also be indirect evidence that the carvacrol density is higher near the surface where polymer chains can be found, whereas the cluster is hollower inside, indicating a preference of drug molecules to associate with the polymer.

The HBPEI system, on the other hand, exhibits a sharper and narrower peak at similar distances, with its width being representative of the average size of observed polymer–carvacrol clusters. A very flat and weak peak observed at around 50 Å is indicative of a certain long-range structure; this is mostly due to the polymer molecules that tend to avoid each other and stay situated far apart, as evidenced by a similar behavior of the HBPEI–HBPEI radial distribution function (Figure 4 right). As a result, carvacrol molecules associated with polymers contribute to the long-range local maximum of the HBPEI–carvacrol rdf curve. Notably, the carvacrol–carvacrol peak (Figure 4 right) overlaps to a large extent with the corresponding HBPEI–carvacrol peak (Figure 4 left), although the wider tail in the direction of longer distances indicates additional contributions from essential oil isolated molecules or clusters.

Finally, by integrating the shell area weighted by the rdf, 4πr^2^g_αβ_(r), over the pair distance, it is possible to determine the average number of molecules of type α surrounding a given molecule of type β. If the local minimum immediately after the first HBPEI–carvacrol rdf peak is taken as a boundary for the coordination shell of the carvacrol around it, this boundary corresponds to about 25 Å. In Figure 4, the peak itself corresponds to 2 carvacrol molecules per HBPEI molecule, whereas the coordination shell accommodates up to 6 or 7 essential oil molecules per polymer. The wide peak observed in the HPG rdf appears to end at about 55 Å, where the majority of existing carvacrol molecules are to be found; in other words, each polymer chain can ‘see’ almost all carvacrol molecules within that distance, since they are all part of a single nanocluster of similar dimensions.

**Figure 5:**
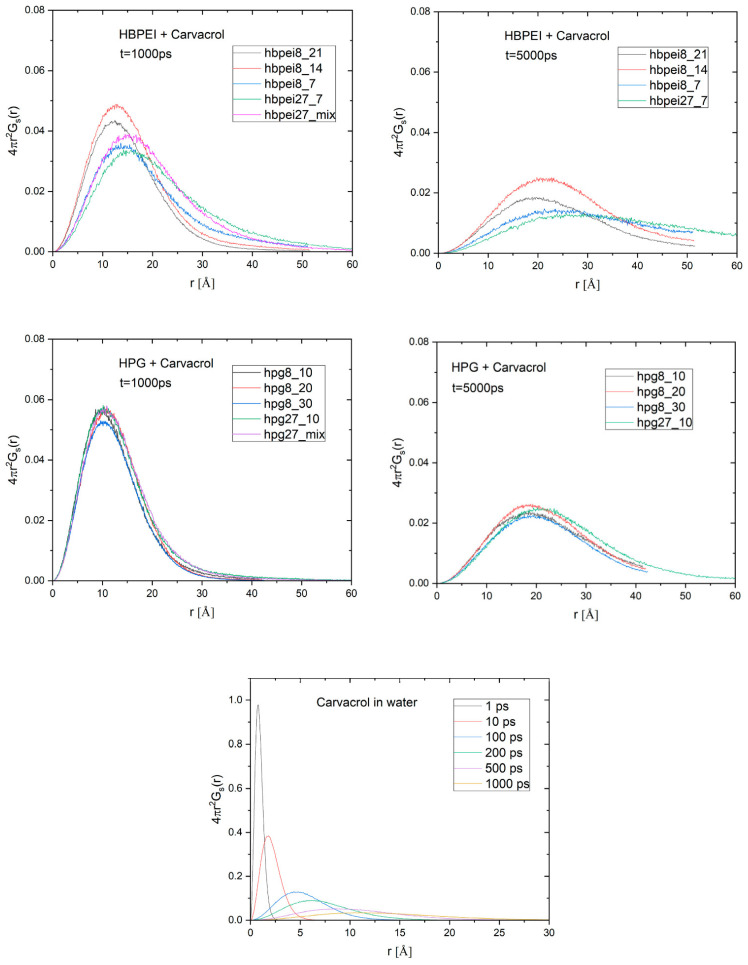
Self-diffusion van Hove functions, 4πr^2^G_s_(r; t), of carvacrol in HBPEI (**top**) and HPG (**middle**) systems for time scales t = 1 ns and 5 ns as functions of the average displacement of carvacrol molecules, *r*. Similar functions for aquatic carvacrol solutions at times between 1 ps and 1 ns are also shown (**lower** panel).

The van Hove functions extend the information provided by the radial distribution functions by providing information related to spatiotemporal correlations of the molecular motion. The drug–drug self-diffusion van Hove functions in Figure 5 reveal that essential oil molecules in HPG systems exhibit nearly identical behavior in terms of positional decorrelation, regardless of the concentration, system size, or coexistence with other ingredients. Thus, the average molecule appears to be displaced by about 12 Å with an uncertainty (based on half-peak heights) of ca. 6 Å within 1 ns; the average displacement increases to a range of 18 to 22 Å with a larger uncertainty of up to 10 Å within 5 ns. HBPEI systems, on the other hand, exhibit a discernible dependence on the carvacrol concentration. The curves describing the lower polymer–carvacrol molecular ratios (i.e., 1/21 and 1/14) are narrower and peak at shorter distances compared to those describing the systems at a higher polymer–carvacrol ratio (1/7). This indicates that when an excess amount of carvacrol is present in the solution, a large number of these molecules organize in clusters, leading to a more confined environment. On the other hand, at higher polymer–carvacrol ratios, a larger percentage of the carvacrol molecules interact with the polymer instead of participating in large clusters, exhibiting wider distributions and peaks at somewhat longer distances.

**Figure 6:**
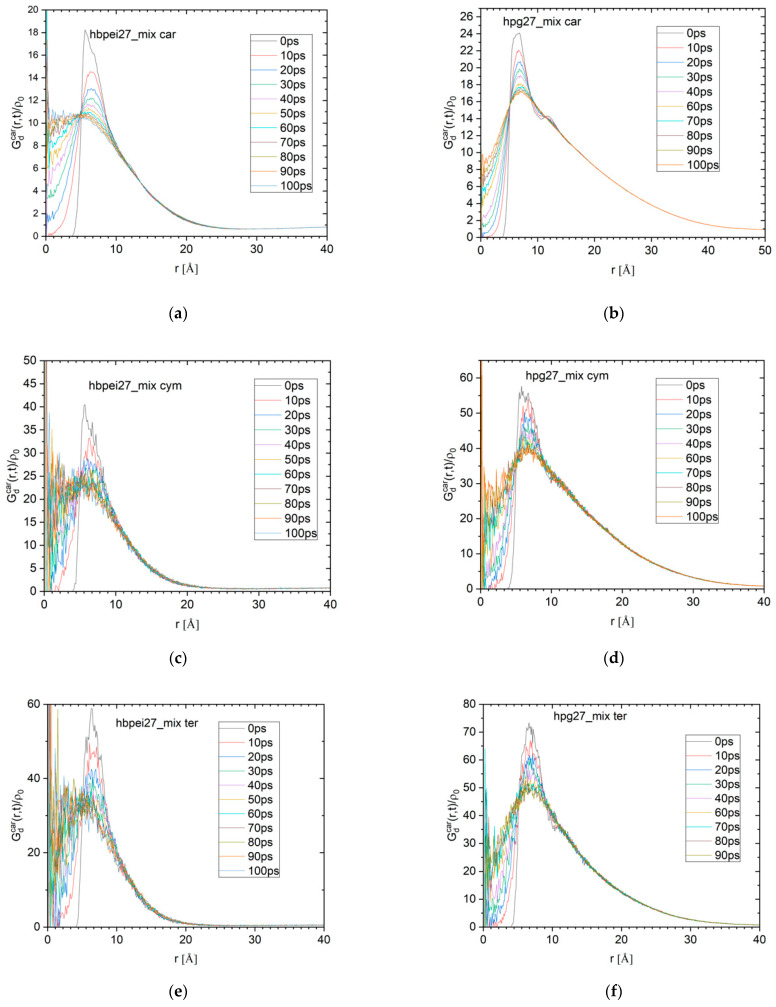

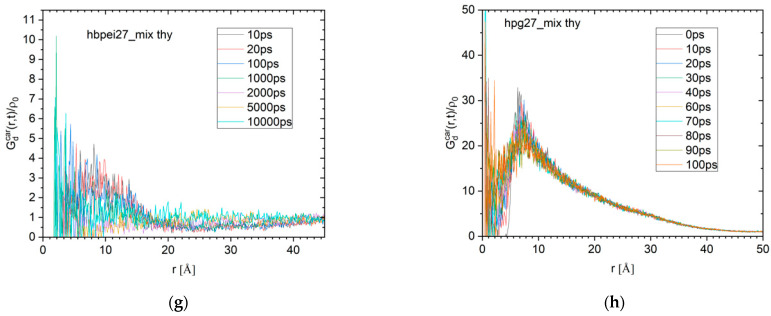
Distinct van Hove functions for (**a**) carvacrol, (**b**,**c**) p-cymene, (**d**,**e**) γ-terpinene, and (**f**,**g**) thymol in (**h**) systems with HBPEI (**left**) and HPG (**right**) and terpenoid mixtures in realistic proportions (27:189:18:15:7 and 27:270:26:22:10, respectively). Statistical noise arises with a smaller number of drug molecules. The distinct van Hove spectra are normalized by the overall density of the examined molecules.

**Figure 7:**
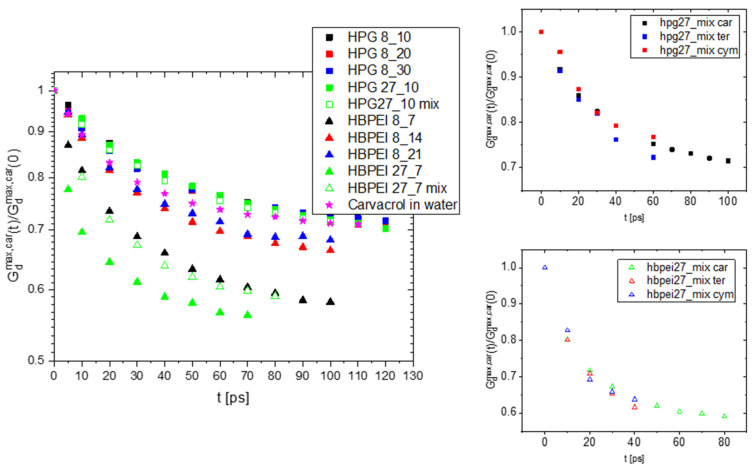
**Left**: Maxima of distinct van Hove functions relative to corresponding values at *t* = 0 for carvacrol in HPG- and HBPEI-containing systems for all concentrations and system sizes studied; data for carvacrol aquatic solution are also included. **Right**: Distinct van Hove functions for γ-terpinene and p-cymene compared with carvacrol in HPG (**top**) and HBPEI (**bottom**) systems.

While the self-diffusion part of the van Hove function monitors the transport characteristics of individual molecules, the distinct part probes the evolution of the local environment (i.e., determined by the relative location of neighbors) around a molecule. This information is of special interest, because of its relationship with the dynamic rearrangements relevant to the clustering process. In Figure 6, curves representing the distinct van Hove function for different time scales are shown for carvacrol in the 27-polymer-molecule systems containing all four essential oil ingredients; similar curves were computed for all other polymer–essential oil systems. The heights of the curves’ peaks relative to the corresponding values for zero time are plotted against the time separation in Figure 7 for all carvacrol-containing systems of all concentrations. Similar data are included for carvacrol in water for comparison. Additionally, data on the polymers with essential oil mixtures are shown in the smaller companion figures. The rate of change of the height of the maxima (i.e., the rate at which the local arrangement of neighboring molecules loses its structural coherence with respect to an initial configuration) is indicative of the time scales characterizing the rearrangement of the local environment at the molecular scale.

Regarding HPG-containing systems, there is practically no dependence on the system size or concentration; the data sets virtually overlap with each other and tend to coincide with the one describing the carvacrol aquatic solution. Apparently, the essential oil’s strong tendency to phase-separate remains more or less unchanged in HPG–carvacrol systems, as shown by the invariably slow decorrelation rate, regardless of the polymer’s presence or the compound’s concentration. The same behavior is observed in HPG systems containing a mixture of essential ingredients (data for thymol are omitted due to their lower statistical significance). Once a single nanodroplet has formed, local rearrangements slow down and are mostly governed by the droplet’s structure, with essential oil occupying the bulk of the cluster and polymer restricted on the surface, causing the similar behaviors across system sizes and concentrations.

HBPEI systems, on the other hand, exhibit significantly faster decorrelation, with the decorrelation time scales increasing with the carvacrol concentration in an apparent agreement with self-diffusion van Hove data. The local environment in systems with a 1/14 or 1/21 HBPEI–carvacrol ratio persists over similar time scales, as in carvacrol or carvacrol–HPG systems, as opposed to the two 1/7 systems, which decorrelate at a faster pace. There is also a certain size dependence, as decorrelation in the large (27-polymer-molecule) system is slower than in the 8-molecule analogue; the other essential oil ingredients exhibit more or less equally slow dynamics as carvacrol in the corresponding 27-polymer-molecule system. With a higher essential oil concentration or larger system size, more drug molecules are present, but also more space is available for them to move freely, as evidenced by the 5 nm length scale and hinted at by the radial distribution functions (Figure 4), thereby resulting in faster local rearrangement dynamics. The presence of more than one essential oil ingredient stabilizes the equilibrium structures and their decorrelation slows down.

**Figure 8:**
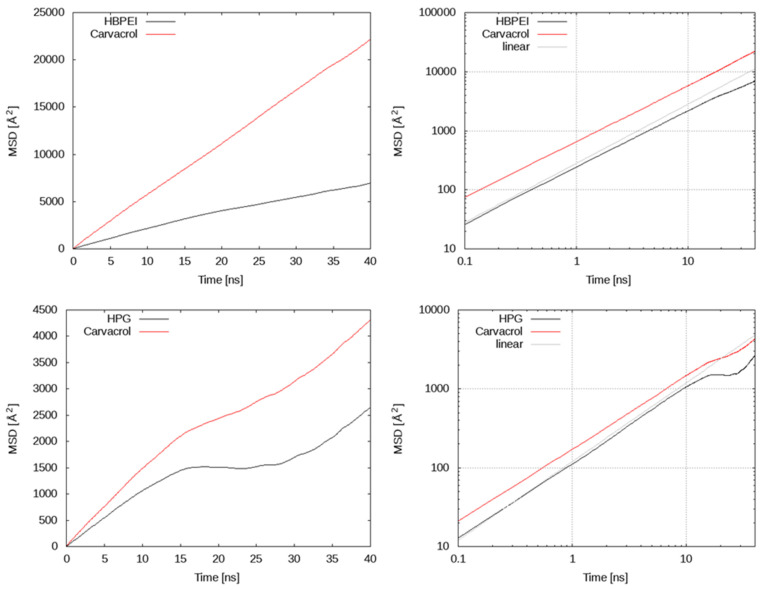
Mean square displacement in linear (**left**) and log–log (**right**) scales of polymer and carvacrol species in 27-polymer chain systems of HBPEI–carvacrol (**top** row) and HPG–carvacrol (**bottom** row).

In Figure 8, the mean square displacement of polymer species is compared to carvacrol for the 27-polymer molecule systems of HPG and HBPEI within a time window of 40 ns; the results were obtained by omitting the first 50 ns of the trajectories. In this time period, clustering had already occurred, and we can safely assume that the system was in a state of dynamic equilibrium (this point is also discussed further below and in the next Section). The linear increase with time clearly breaks down at time scales of about 15 ns for HPG, whereas a close to linear trend persists for longer time scales in the case of HBPEI. The onset of subdiffusive behavior in HPG corresponds to an average displacement of about 35 or 45 Å for the polymer and carvacrol, respectively. This is less than half the average simulation cell length, indicating a kind of trapping of the two species. By comparison, water molecules (curves not shown) have moved over distances of at least an order of magnitude longer. Regarding HBPEI-containing systems, carvacrol molecules diffuse over distances exceeding the simulation cell size, at ca. 12 to 14 nm, within the examined time scales. HBPEI molecules are displaced at a relatively slower rate; still, they do not appear to be inhibited from exploring the entire available space. For both species, the displacements are nearly an order of magnitude larger than in HPG systems. These results are certainly consistent with the visual finding of many free compound and polymer molecules and the presence of small to medium clusters.

**Figure 9:**
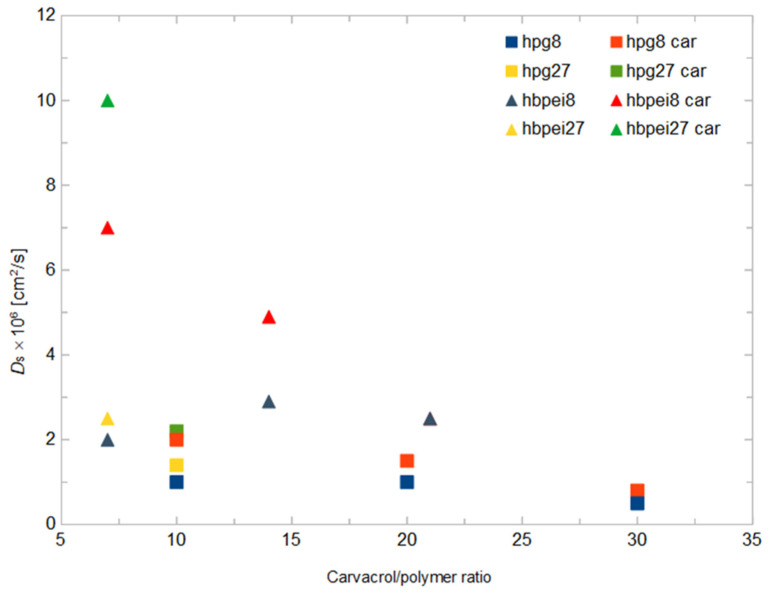
Self-diffusion coefficients (×10^6^ cm^2^ s^−1^) of polymer (yellow and navy symbols) and carvacrol (red and green symbols) in polymer–carvacrol systems, with respect to carvacrol–polymer ratio.

Self-diffusion coefficients of polymer and carvacrol as a function of the drug concentration are shown in Figure 9. Stark differences in diffusivities can be observed between HBPEI and HPG systems for both the polymer and drug compound species. Carvacrol diffuses faster by almost an order of magnitude in low-concentration HPBEI solutions than in HPG ones. HBPEI itself diffuses twice as fast as HPG in low-concentration systems. On the other hand, with increasing carvacrol concentration, the diffusivity levels of the drug compound and polymer tend to converge to the same limiting value. These results are consistent with the visual findings: HBPEI systems contain freely floating molecules and small clusters in dynamic equilibrium, while clusters occur more often and grow in size with concentration, giving rise to slower diffusive dynamics. HPG systems tend to form fewer and larger clusters that tend to coalesce in one droplet, thereby diffusing as a whole and at a slower rate. In both HBPEI and HPG systems, once a stable cluster is formed, it imposes its own diffusive dynamics as a whole on its constituent molecules, resulting in the convergent diffusion coefficients. These results are also partially consistent with van Hove analysis, although the former concerns global dynamics and the latter has to do with local properties, explaining the opposite trends for small and large HBPEI systems at low concentrations.

**Figure 10:**
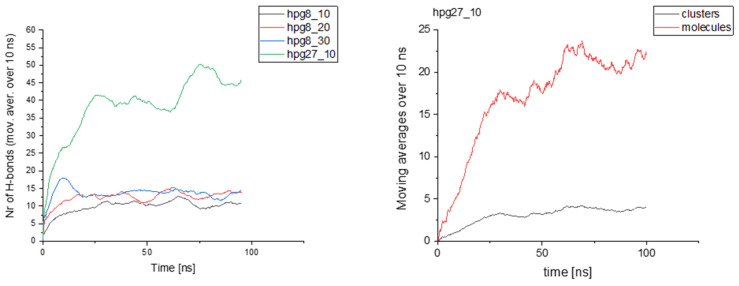
**Left**: Number of carvacrol–polymer hydrogen bonds with time in HPG systems. **Right**: Number of clusters (black) and number of carvacrol molecules found in clusters (red) in a 10:1 carvacrol–HPG 27-polymer-molecule system.

Clustering was found to occur in both HPG- and HBPEI-containing systems, albeit of a different type and to a different extent, and all four essential oil ingredients were seen to participate in the clusters formed. Apparently, the general tendency to form clusters has to do with factors in common among the polymers and terpenoid compounds (amphiphilic character of the former, hydrophobic character of the latter), whereas particular features of the final configuration reflect their differences (hydroxyl groups, hydrogen bonding ability, charged groups in polymer chains). Once essential oil molecules get close to each other and to the polymer chains, they will start to form more hydrogen bonds—if they are generally able to do so—until a steady state is attained; thus, hydrogen bonding, where applicable, can serve as a proxy measure of clustering. As an example, Figure 10 shows the number of carvacrol–polymer hydrogen bonds with time in HPG systems, with the number of clusters and number of carvacrol molecules in clusters in the 27-polymer HPG system with a 1/10 polymer-to-carvacrol ratio (similar curves are obtained for the other systems as well). Generally, the number of hydrogen bonds correlates closely with the numbers of clusters and clustered molecules.

**Figure 11:**
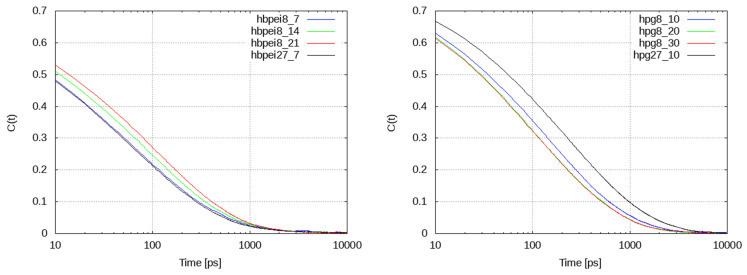
Hydrogen bond autocorrelation functions of HBPEI (**left**)- and HPG (**right**)-containing systems as a function of the carvacrol concentration and system size.

Time autocorrelation functions were calculated by assigning 1 or 0 to the presence or absence of a hydrogen bond at specific time instances:

$$C\left(t\right)=\langle \frac{h\left(t_{0}+t\right)h\left(t\right)}{h{\left(t_{0}\right)}^{2}}\rangle$$

where *h*(*t*) denotes the population of hydrogen bonds formed by a given species at time *t* and the average is taken over all molecules of this species and all time origins, *t*_0_. These functions are shown in Figure 11 for HBPEI and HPG systems. Noticeable increases in decorrelation time scales with drug concentration can be observed in HBPEI systems. Numerical integration of the corresponding autocorrelation functions yields time scales that range between 190 and 222 ps, depending on the carvacrol content. The opposite trend can be observed in HPG systems, with corresponding time scales varying over a wider range, namely from 217 to 285 ps for 8-polymer chain systems or even 395 ps for the 27-chain system. Generally, hydrogen bonding exhibits slower dynamics in HPG than in HBPEI systems.

**Figure 12:**
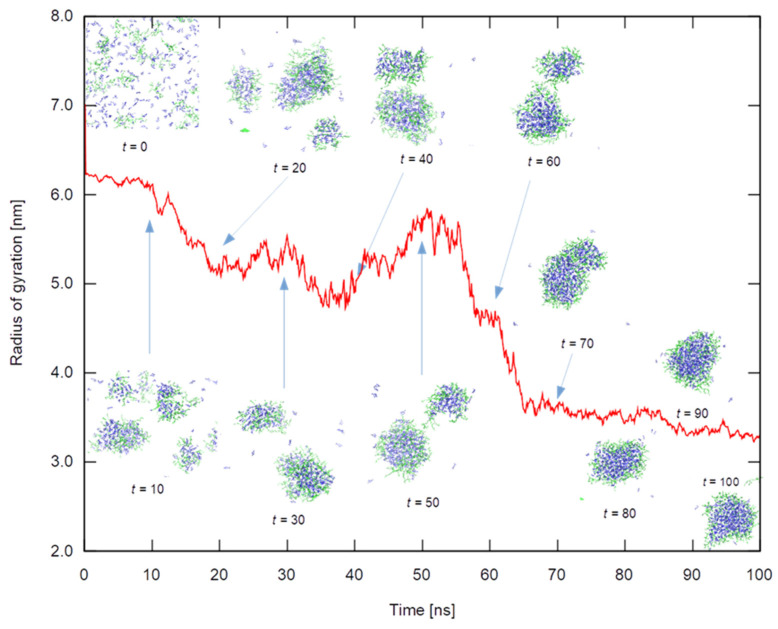
The radius of gyration, in nm, of the swarm consisting of all carvacrol molecules in the corresponding 27-chain HPG system against time. Snapshots of the system (solvent omitted) every 10 ns are also shown.

HPG-containing systems end up with a single cluster containing almost all terpenoid molecules. An alternative quantitative measure of clustering applicable to these systems consists of measuring the size of the swarm that comprises all essential oil molecules (taking periodic conditions into account). Indeed, as clustering evolves with time, less molecules will be free to float independently and explore the entire simulation cell, and more of them will concentrate in a smaller region. Therefore, the swarm size will get smaller with time until equilibrating to a limiting value corresponding to the final cluster size. Figure 12 shows the radius of gyration of the swarm for *all* carvacrol molecules in the 27-chain HPG system as a means to illustrate the cluster formation development. The inset figures depict the stages of the clustering process in which the formation of smaller aggregates can be observed; these in turn will form larger aggregates until coalescing to a single large cluster. It should be noted that the radius was calculated by taking all terpenoid molecules into account, whether clustered or free; the more freely floating molecules around, the larger the radius. This should partially explain why the size of the final cluster is not totally stationary but exhibits a slow decrease in size; this should come about as a combination of internal rearrangements and through the integration of the last freely floating essential oil molecules. Completely analogous dynamics can be observed in the case of the HPG system containing a mixture of all four essential oil ingredients.

**Figure 13:**
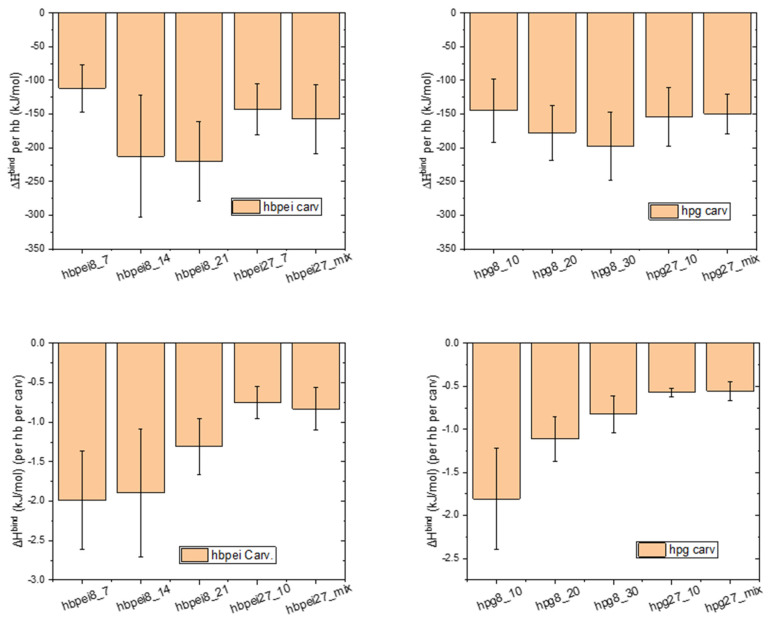
Binding enthalpy per hyperbranched molecule (hb) in the **top** panel and per hyperbranched molecule in the **bottom** panel.

All of the above results elucidate various aspects of the displacement and clustering dynamics, although the underlying mechanisms depend strongly on the interactions among the constituent species. Thus, it is instructive to look at the polymer–drug binding energetics. Τhese calculations were performed using the molecular mechanics–Poisson–Boltzmann surface area method (MM/PBSA) method, as implemented in the g_mmpbsa tool [51]. The parameters used were based on recent relevant works [53,54]. Figure 13 shows polymer–drug binding enthalpy calculations for all polymer–terpenoid systems studied. Error bars are too large to allow for definite conclusions. To the extent that the computed averages represent actual trends, larger drug concentrations imply stronger polymer–terpenoid associations (Figure 13, top). On the other hand, single polymer–terpenoid pairs appear to weaken with an increasing number of terpenoid molecules in the system, probably due to competition with their own (terpenoid–terpenoid) tendency to associate.

## 4. Discussion

The spatiotemporal scales in which the subdiffusive motion takes effect in HPG systems is consistent with the formation of a single large cluster. The associated molecules are restricted to move together as a single entity. Occasionally, carvacrol or polymer molecules are released and wander around for several nanoseconds before reintegrating with the polymer–carvacrol nanodroplet. On the other hand, HBPEI systems adopt more diffuse or network-like configurations consisting of smaller clusters and free polymer and essential oil molecules in dynamic equilibrium. Not only do free species move over longer spatiotemporal scales but the clusters formed are also faster to diffuse in comparison with the HPG single nanodroplet—although they do have an inhibiting effect on their own constituent molecules, which explains such findings as the sublinear mean square displacement.

Size-dependent effects that arise in several calculations merit some discussion with regards to the properties examined. With larger system sizes and higher numbers of polymer and terpenoid molecules, longer time and length scales emerge, but also more associations in terms of cluster size and composition are possible, implying a complex landscape of structural and dynamic properties. For instance, radial distribution functions of HBPEI systems hint at a fragile yet constantly present long-range order characterized by a 5 nm length scale, consistent with the visual finding of small clusters and freely floating polymer and terpenoid molecules in dynamic equilibrium, forming a dynamic network structure. The absence of this length scale in smaller systems deprives molecules from diffusing freely for long enough prior to associating with other terpenoid or polymer species, resulting in relatively smaller self-diffusion coefficients (at low terpenoid concentrations); relatively slower local rearrangement dynamics, as evidenced by the van Hove analysis; and on average stronger polymer–drug interaction pairs. Polymer diffusivity is slower, as expected, and less affected by concentration, thereby also controlling terpenoid transport and local dynamic properties to the extent that polymer–drug associations become more important with increasing concentration. On the other hand, HPG systems exhibit a simpler behavior characterized by the dominant role of the large nanoclusters formed at the final stages of our simulations; this role generally consists of slowing down the system dynamics both locally (e.g., van Hove, hydrogen bond autocorrelation) and globally (mean square displacements).

The hydrogen bond decorrelation time scales show that the essential oil fractional content is a strong determinant of clustering in HPBEI systems, regardless of the system size. Higher essential oil concentrations correlate with slower hydrogen bonding dynamics, which should be a consequence of a stronger tendency of drug ingredients to phase-separate and trap more molecules in emerging small clusters. On the other hand, HBPEI molecule tend to avoid each other due to their charges, which are only partially screened as the counterions prefer to remain dispersed in solution, most probably due to entropic reasons. HPG systems, on the other hand, lead to the formation of larger and more stable clusters where drug and polymer molecules have less freedom to move relative to their neighbors, thus allowing hydrogen bonds to last longer. The system size appears to be a stronger determinant than concentration, here. In our opinion, this is more than a mere simulation artifact; it is an indication of the strong tendency of the essential oil ingredients to phase-separate within the examined time scales, with the polymer playing a secondary or companion role in the process.

From the above analysis, it may be concluded that polymer–drug association is favored by low drug concentrations in HBPEI so that the isolated and mutually repelling polymers can compete with the drug compounds’ tendency to phase-separate and form pure essential oil clusters. This is not an issue in HPG systems, as the neutral HPG molecules do not repel each other, meaning they can easily attach to the essential oil clusters and form micelle-like structures. However, the formation of single clusters in all systems examined suggests that essential oil phase separation is a strong and prevalent tendency that could eventually extend to meso- and macroscopic scales with the polymer occupying the interfacial region between drug compounds and solvent. Thus, low polymer–drug proportions are recommended for both systems to achieve the formation of nanoscale water-soluble clusters; strong agitation prior to administration would also be advisable.

Finally, it should be noted that HPG systems tend to form micelle-like structures with polymer on the outside and terpenoid inside, whereas HBPEI clusters consist mainly of single or several polymer molecules surrounded by drug molecules. The configuration adopted by the latter systems is probably due to the repulsions among the partially shielded charges of the polymer. Whether such an effect would persist for HBPEI–drug clusters in actual conditions of interest, as found in the blood circulatory system, is uncertain. Since HPG polymers are not charged and are less susceptible to interactions with other species, it may be assumed that HPG–drug micelles could also be relatively more stable when tested in real-life applications.

## 5. Conclusions

We have employed molecular simulation methods to study four candidate drug compounds of the terpenoid family (carvacrol, thymol, p-cymene, γ-terpinene) that can be found in species of Greece’s endemic flora, together with two candidate drug carrier species, hyperbranched poly(ethylene imine) and hyperbranched poly(glycerol). We have simulated aquatic systems of polymer–terpenoid combinations at a range of concentrations and two different system sizes to look at the ability of the polymers to associate with the terpenoids and solubilize them, so as to render them capable of being administered as pharmaceutical drugs. The role of the amphiphilic polymers studied is two-fold: on one hand, they form water-soluble clusters that could be transported in the blood circulating system; on the other hand, they will potentially allow the clusters to integrate with cell membrane and release their drug content therein.

It was found that both polymer species were capable of associating with terpenoids, although the configurations adopted by the two kinds of systems differed substantially. Thus, HBPEI systems adopted diffuse network-like structures of small clusters together with freely floating polymers in dynamic equilibrium, whereas HPG systems tended to form larger nanodroplets containing almost all terpenoid molecules in the simulated systems. The terpenoid tendency to phase-separate seemed to be the dominant factor governing the clustering process, with polymers playing the role of the nucleation seed. Once associated, the terpenoid dynamics were restricted by the dominant presence of the polymers, and above all the clusters formed.

It has, therefore, been verified that the two hyperbranched species studied are promising drug carriers. The collected evidence also suggests that it is safer to try to lower the terpenoid-to-polymer proportions to prevent the drug molecules from phase separation. Most probably, HPG should be preferred over HBPEI, as the former adopts micelle-like structures that protect their drug content and are more or less unaffected by the presence or absence of other charged species in their immediate environment.

## Figures and Tables

**Table 1:** Size and composition of terpenoid–polymer aquatic solutions. The numbers of the rightmost column shown in italics, denote the total number of atoms in each model.

System Key to Figures	Kind	Molecules	Atoms/Molecule	Total Number of Atoms
Carvacrol car	carvacrol	213	25	5325
	Water	9403	3	28,209
				*33,534*
Thymol thy	thymol	209	25	5225
	Water	8526	3	25578
				*30,803*
γ-Terpinene ter	γ-terpinene	245	22	5390
	Water	8207	3	24,621
				*30,011*
p-Cymene cym	p-cymene	243	24	5832
	Water	8279	3	24,837
				*30,669*
Mixture mix	carvacrol	1704	25	42,600
	γ-terpinene	144	22	3168
	p-cymene	168	24	4032
	thymol	64	25	1600
	Water	75,224	3	225,672
				*277,072*

**Table 2:** Size and composition of terpenoid–polymer aquatic solutions. The numbers of the rightmost column shown in italics, denote the total number of atoms in each model.

System Key to Figures	Kind	Molecules	Atoms/Molecule	Total Number of Atoms
HBPEI (1:7) hbpei8_7	HBPEI	8	355	2840
	carvacrol	56	25	1400
	Water	34,040	3	102,120
	Cl^–^ ions	112	1	112
				*106,472*
HBPEI (1:14) hbpei8_14	HBPEI	8	355	2840
	carvacrol	112	25	2800
	Water	34,040	3	102,120
	Cl^–^ ions	112	1	112
				*107,872*
HBPEI (1:21) hbpei8_21	HBPEI	8	355	2840
	carvacrol	168	25	4200
	Water	34,040	3	102,120
	Cl^–^ ions	112	1	112
				*109,272*
HBPEI (1:7) 27 chains hbpei27_7	HBPEI	27	355	9585
	carvacrol	189	25	4725
	Water	114,885	3	344,655
	Cl^–^ ions	378	1	378
				*359,343*
HBPEI (1:7) 27 chains hbpei27_mix	HBPEI	27	355	9585
	carvacrol	189	25	4725
	p-cymene	18	24	432
	γ-terpinene	15	26	390
	thymol	7	25	175
	Water	114,885	3	344,655
	Cl^–^ ions	378	1	378
				*360,340*
HPG (1:10) hpg8_10	HPG	8	210	1680
	carvacrol	80	25	2000
	Water	17,752	3	53,256
				*56,936*
HPG (1:20) hpg8_20	HPG	8	210	1680
	carvacrol	160	25	4000
	Water	17,752	3	53,256
				*58,936*
HPG (1:30) hpg8_30	HPG	8	210	1680
	carvacrol	240	25	6000
	Water	17,752	3	53,256
				*60,936*
HPG (1:10), 27 chains hpg27_10	HPG	27	210	5670
	carvacrol	270	25	6750
	Water	59,913	3	179,739
				*192,159*
HPG (1:10) 27 chains hpg27_mix	HPG	27	210	5670
	carvacrol	270	25	6750
	p-cymene	26	24	624
	γ-terpinene	22	26	572
	thymol	10	25	250
	Water	59,913	3	179,739
				*193,605*

## Data Availability

Not applicable.
